# Ketamine-Dexmedetomidine Sedation in the Management of a Child With Generalized Refractory Myasthenia Gravis and Respiratory Insufficiency: Case Report

**DOI:** 10.7759/cureus.47344

**Published:** 2023-10-19

**Authors:** Beatriz V Grenho, Filipa Carioca

**Affiliations:** 1 Anesthesiology, Hospital Garcia de Orta, Almada, PRT; 2 Anaesthesiology, Centro Hospitalar Universitário de Lisboa Central, Lisbon, PRT

**Keywords:** respiratory insufficiency, procedural sedation and analgesia, ketamine-dexmedetomidine, myasthenia gravis (mg), pediatric anaesthesia

## Abstract

Myasthenia gravis (MG) is a rare autoimmune disease characterized by muscle weakness and fatigability that can be generalized with respiratory muscle involvement. The anaesthetic management of these patients poses significant challenges not only because of the nature of the disease and its possible interactions with anaesthetic drugs but also because of the risk of developing a life-threatening myasthenic crisis. We report the case of a child with generalized refractory MG and pneumonia, worsening his chronic respiratory insufficiency, proposed for an urgent thoracocentesis and chest drain placement, that was successfully managed under sedation with ketamine and dexmedetomidine, allowing to avoid a general anaesthesia, preserve the patient’s respiratory drive, and protect his airway. The present report highlights the potential applicability of this novel drug association in MG patients, regarding its safety and efficacy in maintaining respiratory drive and haemodynamic stability in these patients.

## Introduction

Myasthenia gravis (MG) is a rare autoimmune disease characterized by the presence of antibodies against the postsynaptic acetylcholine receptor (AChR) at the neuromuscular junction. This disorder has an annual incidence of 7 to 23 new cases per million and has a bimodal distribution, with a peak in the first three decades of life and the other between the sixth and eighth decades [1]. Patients display weakness and fatigability of skeletal muscles that vary in clinical form and severity, ranging from the focal weakness of the ocular and extraocular muscles, resulting in ptosis and diplopia, to generalized MG, involving respiratory, bulbar, and limb muscles, ensuing dysarthria, dysphagia, and respiratory insufficiency. Myasthenic crises are usually precipitated by physiological stress, including surgery and infection, and may result in the need for mechanical ventilation [1]. Management of MG can be challenging and includes symptomatic therapy with acetylcholinesterase inhibitors, thymectomy, chronic immunosuppression with azathioprine, and, in refractory cases, monoclonal antibodies such as rituximab and eculizumab [2].

The anaesthetic management of these patients poses significant challenges and is of paramount importance in the maintenance of delicate homeostasis and the prevention of life-threatening complications related to the disease.

In the present report, we discuss the anaesthetic management of a child with refractory generalized MG with pneumonia and pleural effusion who was submitted to urgent thoracocentesis and chest drain placement under only sedation with ketamine and dexmedetomidine (ketodex). Following the literature review, this is the first reported case of the use of this novel drug combination for sedation in a paediatric patient with generalized MG.

## Case presentation

A 14-year-old boy, 81 kg, presented to the hospital with fever, malaise, and chest pain, being diagnosed with pneumonia and left pleural effusion. Antibiotic therapy was started, and he was proposed for an urgent thoracocentesis, chest drain placement, and peripherally inserted central catheter (PICC) insertion. The patient had a previous medical history of generalized refractory type IVb MG (by the Myasthenia Gravis Foundation of America (MGFA) classification) diagnosed two years prior and medicated with eculizumab and pyridostigmine 450 mg/day, osteopenia secondary to corticoid therapy, iatrogenic phrenic nerve paralysis after thymectomy in 2021, chronic respiratory insufficiency under nocturnal non-invasive ventilation, and was overweight.

**Figure 1:**
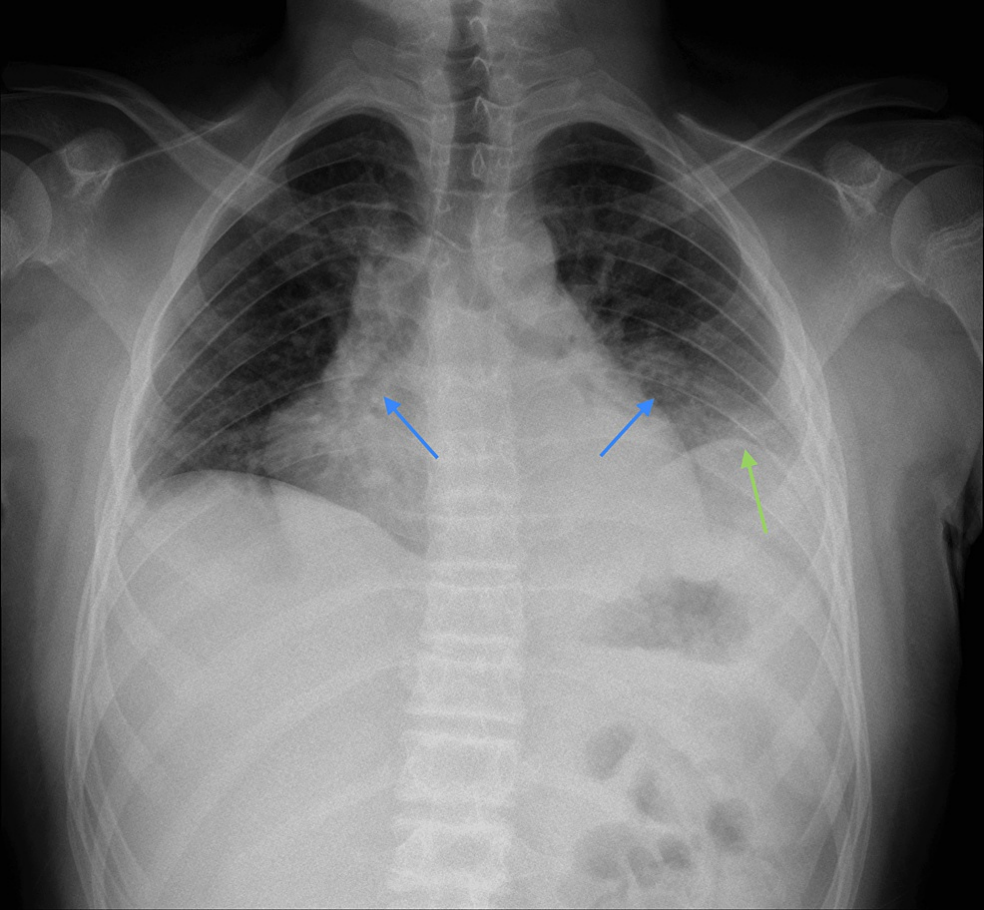
Preoperative X-ray Blue arrows: bilateral infiltrate, green arrow: left pleural effusion.

On examination, he was tachypneic (respiratory rate of 22 breaths per minute), had 96% saturation under 2 L/min oxygen supplementation, and, although cooperative, got easily tired when speaking. He also displayed bilateral ptosis, looked expressionless, and had discrete proximal limb weakness. On auscultation, the murmur was globally decreased and absent on the inferior third of the left hemithorax. Blood analysis showed leucocytosis with neutrophilia and an increased C-reactive protein (86.8 mg/dL) with normal cardiac biomarkers. The electrocardiogram (EKG) showed sinus tachycardia with no evidence of pericarditis or acute coronary syndrome. On the chest X-ray, a left pleural effusion and bilateral infiltrate were evident (Figure 1).

The patient had no dysarthria, no history of dysphagia, or any co-existing autoimmune disorders. Before coming to the operating room (OR), his usual dose of pyridostigmine was administered. On arrival at the OR, American Society of Anesthesiologists (ASA) standard monitoring was applied, and his saturation was 96% under 2 L/min of oxygen, his heart rate (HR) was 105 bpm, and his blood pressure (BP) was 100/52 mmHg. A nasal cannula with capnography monitoring was placed. To start sedation, 1 mcg/kg of dexmedetomidine and 1 mg/kg of ketamine were slowly administered over a period of 10 minutes. The surgeon proceeded with the infiltration of the thoracocentesis site with 5 mL of ropivacaine 0.2%.

Afterwards, an infusion of dexmedetomidine 1 mcg/mL with ketamine 1 mg/mL was started at 1 mL/kg/h and was progressively titrated (reaching a maximum of 1.6 mL/kg/h) according to the patient’s response and the stimulus of the procedure. Extra boluses of ketamine (10 mg) were given, when necessary, for a total of two. Paracetamol 1 g, ketorolac 30 mg, and ondansetron 4 mg were also administered during the procedure.

During the intra-operative period, the patient tolerated the entire procedure and preserved spontaneous ventilation and airway reflexes throughout, with adequate saturations and end-tidal carbon dioxide concentration. Hemodynamic stability was also maintained, with only one decline of HR to 60 bpm after the first administration of ketodex, and no effect on the BP. There were also no other side effects from ketamine and dexmedetomidine, such as hypersalivation, the emergence agitation, or hypotension.

**Figure 2:**
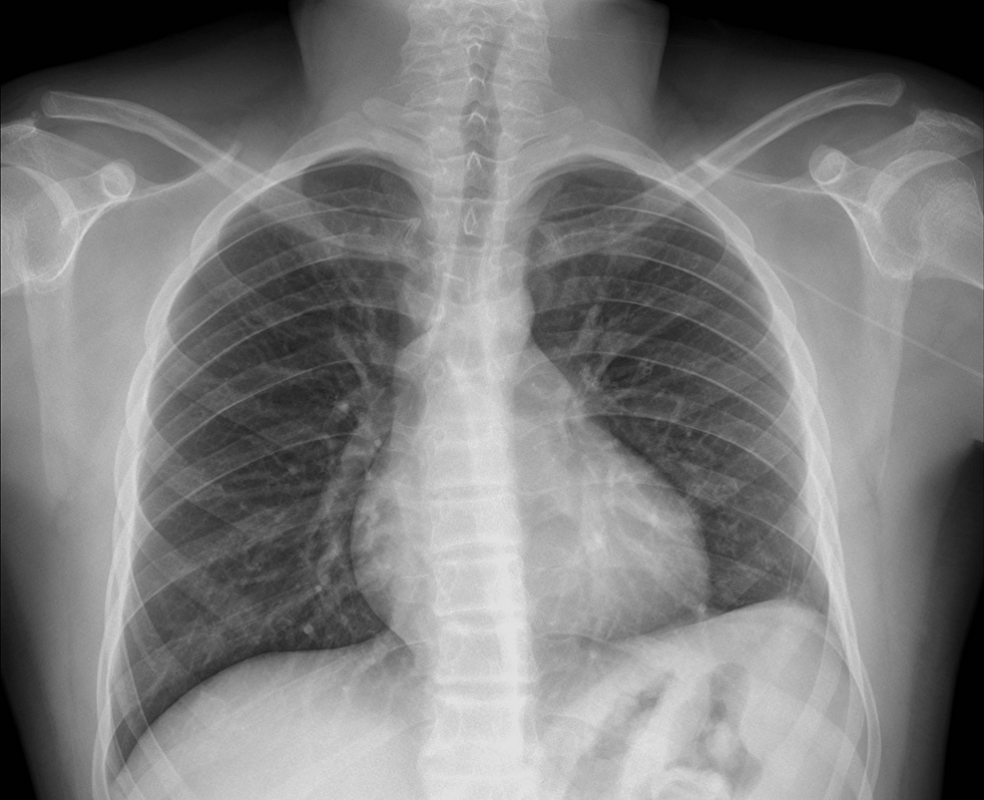
X-ray at discharge

Towards the end of the procedure, the ketodex infusion was titrated down and subsequently stopped. The patient, already awake, painless, and less tachypneic (respiratory rate under 18 breaths per minute), was transferred to the postanaesthetic care unit (PACU), where he remained closely monitored for two hours. After reducing the oxygen supplementation to the preoperative flow, he was transferred to the ward, and the pyridostigmine was recommenced. The patient was discharged home on day 14 after improvement of clinical and inflammatory parameters and completion of an antibiotic cycle (Figure 2).

## Discussion

MG presents significant challenges to the anaesthetist, not only because of the nature of the disease itself, the possible interactions between the disease and its treatment, and the anaesthetic drugs, but also because of the risk of developing a life-threatening myasthenic crisis, precipitated by physiological stress such as infection and surgery [3].

In the preoperative period, it is essential to document the severity of weakness and muscle groups affected, mainly focusing on respiratory and bulbar function, as well as to exclude other coexisting autoimmune and inflammatory muscle disorders, like myocarditis [2-3]. Our patient had a documented chronic respiratory insufficiency but no history of bulbar symptoms that could increase the risk of aspiration or other autoimmune diseases. Myocarditis and acute coronary syndrome were also excluded.

Many scoring systems have been developed to predict the risk of a myasthenic crisis after surgery, with the most important risk factor being the presence of previous respiratory problems or co-existing lung disease [4]. Regarding anticholinesterase medication, it is recommended that the usual doses be administered preoperatively, especially in severe MG cases, to help prevent the onset or worsening of respiratory weakness [3-5].

In the intraoperative period, the main anaesthetic goals are to avoid prolonged drug effects on respiratory muscles that would prevent a rapid recovery after surgery [6]. Therefore, care must be taken with the administration of drugs that may worsen MG. It is known that these patients are resistant to depolarizing neuromuscular blocking agents (NMBA) and have increased sensitivity to nondepolarizing NMBA, so intubation, if necessary, should be accomplished without NMBA when feasible [3]. Inhalation anaesthetics, because of their reduction in neuromuscular transmission, may make it possible to avoid NMBA in MG patients [4]. Although there seems to be no clinical evidence [4], acetylcholinesterase inhibitors could impair the hydrolysis of ester local anaesthetics, and so we chose an amide local anaesthetic for the infiltration of the surgical site.

Considering the generalized refractory MG with associated chronic respiratory insufficiency displayed by our patient and its concomitant increased risk of a myasthenic crisis after surgery, avoiding general anaesthesia and NMBA administration appeared to be the most adequate and cautious option. To reach this goal, and given its proven safety and efficacy in the paediatric population [7], sedation using ketodex and local anaesthesia were administered. This novel drug association allowed us to keep the patient sedated and tolerating the procedure while preserving his respiratory drive and protecting his airway, which was of the utmost importance in this case.

The association of ketodex has proven to be more efficacious and safe than the use of each drug alone because, on the one hand, there is a synergy in their sedative effects, and on the other hand, there is a balance of each drug’s side effects, with dexmedetomidine alleviating ketamine-induced tachycardia, hypersalivation, and emergence agitation and ketamine reversing the bradycardia and hypotension induced by dexmedetomidine [7]. This was demonstrated in the present case, in which we were able to maintain adequate sedation throughout the procedure, with only a brief decrease in HR following the first ketodex dose, always maintaining BP and hemodynamic stability.

Finally, a regional anaesthesia technique, such as serratus plane or intercostal nerve block, was not performed due to the potential risk of further deterioration of the patient’s respiratory insufficiency in case of a complication of the technique, such as pneumothorax or respiratory muscle weakness. Instead, we opted for local anaesthetic infiltration of the incision site by the surgeon, which proved to be adequate.

While a literature review revealed one case where ketodex was used for induction of general anaesthesia in a MG patient [8], the present report seems to be the first of a MG paediatric patient successfully managing under sedation with only ketodex.

## Conclusions

MG is a rare autoimmune disease that may be generalized by presenting respiratory insufficiency and bulbar symptoms. The main anaesthetic goals in the management of these patients are to avoid prolonged drug effects on respiratory muscles that would preclude a rapid recovery after surgery and to avoid the development of a life-threatening myasthenic crisis. The present case highlights the potential future applicability of ketodex in MG, allowing the avoidance of general anaesthesia and NMBA and decreasing the risk of developing a myasthenic crisis.
